# User informed design of oral dispersible strips (ODS) to deliver pediatric antiretroviral therapy in Kenya: A mixed methods evaluation of product preferences and acceptability

**DOI:** 10.1371/journal.pone.0334310

**Published:** 2025-10-27

**Authors:** Sarah Finocchario-Kessler, May Maloba, Catherine Wexler, Fred Were, George Mugendi, Yvonne Mbithi, Zachary Nicolay, Michael Hageman, Shadrack Babu, Nicodemus Maosa, Kara Knapp, Gregory Thomas, Sally Maliski, Edward Maliski

**Affiliations:** 1 University of Kansas Medical Center, Department of Family Medicine & Community Health, Kansas City, Kansas, United States of America; 2 Global Health Innovations, Nairobi, Kenya; 3 University of Nairobi, Nairobi, Kenya; 4 Oak Therapeutics, Inc., Lawrence, Kansas, United States of America; 5 University of Kansas, Department of Pharmaceutical Chemistry, Biopharmaceutical Innovation & Optimization Center, Lawrence, Kansas, United States of America; 6 University of Kansas, Center for Design Research, Lawrence, Kansas, United States of America; Kohat University of Science and Technology (KUST), PAKISTAN

## Abstract

**Background:**

Innovation to streamline the administration of pediatric antiretroviral therapy to infants and small children is urgently needed. The objective of this study was to assess user acceptability of key characteristics of prototype placebo oral dispersible strips (ODS) that would contain a pediatric HIV regimen of abacavir + lamivudine+ dolutegravir (ALD), and the extent to which they would address current barriers to ART adherence.

**Methods:**

We conducted 7 focus group discussions with 64 caregivers across three hospitals; 3 FGD with 25 pediatric healthcare providers; and interviews with 25 children living with HIV (6–10 years). All FGD and interviews were audio recorded, translated, transcribed verbatim, and coded for a-priori and emergent themes. We evaluated the acceptability of key characteristics of placebo ALD-ODS (e.g.,flavor, size, dose, dissolution time, colors, and packaging) among caregivers, providers, and children using both qualitative and quantitative methods. Data were integrated and triangulated to optimize understanding of stakeholder feedback.

**Results:**

Adult participants preferred the neutral “sweet” flavored ALD-ODS which was found highly acceptable among children. Caregivers advocated for a single strip per dose and single dose per day for convenience and to reduce stress of administration. In general, strip size and dissolution time was deemed acceptable, and caregivers stated that a larger size would be acceptable to accommodate a single strip per dose. Providers liked aligning the color of ALD-ODS and its packaging to different weight-based dosing to facilitate dispensing and improve accurate administration among multiple children. Most children stated that they would prefer ALD-ODS to their current regimen given the sweet taste and easy dissolution without the need for water. The competitive advantages of ALD-ODS over traditional pediatric ART options were ease of administration- particularly for infants and small children, a sweet taste, and discretion that protected privacy.

**Conclusion:**

Providers and caregivers believed the ODS prototypes held great promise to ease ART administration to infants and young children which would improve medication adherence.

## Introduction

While Kenya has made significant gains in preventing perinatal HIV transmission, progress between 2019 and 2023 has slowed [1]. Gaps in prevention and treatment attributed to late engagement or early disengagement from care, stigma, and system level interruptions to drug supplies and viral load monitoring contribute to over 4,000 infants born with HIV annually [1,2]. Pediatric antiretroviral therapy (ART) regimens continue to pose significant challenges to adherence [3–5]. Historically, HIV prevention and treatment options for infants and small children have been improvisational versions of adult regimens that relied on partial doses being crushed, dissolved, and administered to children [4,6].

Recognizing that a drug’s effectiveness is dependent on a patient’s ability to take the medication in the prescribed manner, greater emphasis has been placed on patient centric pharmaceutical drug product design in the past decade [7]. Innovations in pediatric ART include infant syrups with improved palatability [8,9], drug containing sprinkles that can be added in soft food (e.g., porridge) [10,11], or most recently, granules and tablets that are dispersible in water [12,13]. These advances, however, still require cumbersome administration, potable water, and full consumption of the liquid with dispersed or dissolved drug [4,14].

The goal of patient centric design is to ensure that characteristics of the product (e.g., route of administration, dosing frequency, dosage form, taste, appearance, packaging, etc) align with characteristics of the target patient population (e.g., age, access, swallowing ability, dexterity, etc) [15], and provide a competitive advantage to existing options. One effort to simplify administration and adherence to pediatric ART is loading oral dispersible strips (ODS) with weight-based doses of three antiretrovirals. ODS are mucoadhesive and dissolve in the oral cavity to release medication. ODS have been used to administer other pediatric doses of medications to treat epilepsy, nausea and vomiting, constipation, and allergies [16,17], but the quantity of active pharmaceutical ingredient required for a therapeutic dose of ART is high and has posed a barrier to using ODS for pediatric or adult HIV treatment. Scientists at Oak Therapeutics established proof of principle demonstrating bioavailability and bioequivalence of an ODS containing three antiretroviral drugs in the approved pediatric HIV regimen; abacavir, lamivudine, and dolutegravir (ALD) ODS [18]. The ALD-ODS are mucoadhesive, dissolve within 30–45 seconds, have a sweet taste, and can be color coded to indicate weight-based doses [18,19].

Guided by Human Centered Design principles [7,20,21], this multidisciplinary team of researchers aimed to assess the acceptability, feasibility, and extent to which prototype ODS characteristics align with the needs and requirements of key users [22–24]. The study builds upon our formative research with caregivers and providers to assess persistent challenges in ART administration and adherence and optimal product characteristics of alternative delivery strategies for ART to maximize acceptability, utility, and sustained drug adherence among children. Key challenges included the bitter taste of the syrups which led to child anxiety and refusal to take the medication, and complex dosing, administration, and storage of the drugs that compromised privacy [3]. Stakeholders consistently recommended prioritizing a sweet taste, rapid dissolution, discreet administration, and easy storage of the ODS [25]. Guided by these data the pharmaceutical development team at Oak Therapeutics created multiple ART-ODS prototypes reflecting the preferences and recommendations for a patient and caregiver friendly product. In this study, stakeholders (caregivers, providers, and children) qualitatively described and quantitatively evaluated the acceptability and utility or placebo ALD-ODS prototype characteristics to inform final product design for delivery of pediatric ART.

## Methods

This study was implemented in Kenya between February 5^th^ and May 8^th^, 2023 at three government hospitals; 2 in the western region and 1 in the coastal region of the country. Three study team members trained in qualitative interviewing (MM, SB, NM) conducted a total of 7 focus group discussions (FGD) with caregivers and relevant health care providers and 25 semi-structured interviews with children living with HIV aged 6–10 years who were accompanied by their caregiver. Mentor mothers, with established care relationship with patients and caregivers, helped identify eligible participants. The study team reached out to each potential participant (or guardian of the eligible child participant) to explain the research objectives and assess interest in participating. Those interested in participating (or their guardian) signed a written informed consent. Guardians of children were invited to accompany their child during the interview and all participating children provided written assent prior to the interview. All FGDs and interviews were conducted in a private room in the health facility and lasted approximately 75 minutes and 30 minutes, respectively. Participants could opt to taste the placebo ODS that contained no active pharmaceutical ingredients. Procedures and data collection tools were reviewed and approved by Institutional Review Boards at the University of Kansas Medical Center and the University of Nairobi.

Questions from the FGDs guides qualitatively evaluated the acceptability of key ODS characteristics including flavors, sizes, strips per dose, dissolution time, colors and color-coding strategy, and packaging options. Interview guides sought to understand children’s strategies and barriers to taking their current medications and elicited feedback on the ALD-ODS prototypes’ taste, ease of use, and preference for medicine administration (e.g., current formulation [tablets] or ODS). A brief demographic survey was completed prior to the FGD. Participants completed a post-FGD assessment to rank each of the following ODS characteristics outlined in Table 1. The assessment utilized visual images to enhance clarity and understanding of the ODS characteristics being evaluated and a rating scale with a combination of face emojis (angry to extremely happy) and stars to depict acceptability/satisfaction. The participants ranked the flavors (Mango, Sweet, Strawberry, and Orange) in order of their preference, with 4 indicating the most preferred flavor and 1 indicating the least preferred. All other ODS characteristics were ranked 1–5, with 5 indicating the most preferred option or scenario and 1 indicating the least preferred. Children were offered the opportunity to taste the sweet flavored ODS (top choice among adults) and provide general feedback, rather than rank ordering all flavors.

**Table 1:** Ranked characteristics of the ALD-ODS Characteristics and scoring systems used.

Characteristic	Description/Notes	Options	Scoring
ODS Flavors	Ranked by all participants	Sweet	1-4, with 1 being least and 4 being most
	Ranked by all participants	Mango	
	Unavailable to n = 46 participants	Orange	
	Unavailable to n = 46 participants	Strawberry	
ODS Size	1.25“ x 0.47” rounded edge	Very small	1-5, with 1 being least and 5 being most acceptable
	1.25“ x 0.67” rounded edge	Small	
	1“ x 1” square	Medium	
Strips per dose	Dependent on child age and regimen		
Dissolution time	Measured from time placed in mouth to time completely dissolved. Impacted by strip size		
Colors and color coding	Purple, blue, yellow, orange, pink, green: Dependent on weight band		
Packaging: ease of opening	Peel apart from corner	Peel	
	Tear across top	Tear	
Doses per day (not ranked)	Dependent on child age and regimen, determined by dosing guidelines, not user preferences		Not ranked

### Analyses

All FGDs and interviews were audio recorded, translated, and transcribed verbatim. FGD facilitators met routinely to discuss emerging themes to gauge saturation of data. Two study team members (CW, KK) members trained in qualitative research methods developed the structure of the codebook. Transcripts were then coded for a-priori and emergent themes using Excel, and exemplary quotes were identified for each theme. This process was repeated for caregivers, providers, and children. The participant demographic surveys and post-FGD surveys were analyzed using descriptive statistics. The evaluation of ALD-ODS flavor was not consistent across FGD. Some participants did not get to test all the flavors and only compared Sweet and Mango (n = 54) while others tested all 4 of the flavors (n = 34). After eliminating the two flavors consistently ranked as least favorite from consideration, mean rankings for the sweet and mango group were adjusted to reflect a 4-point scale and we used pairwise ranking among all participants to determine preferences between the top two flavors: sweet and mango.

The mean rank and standard deviation of each ODS characteristic was calculated and integrated throughout the qualitative data, providing a point of triangulation for feedback on the prototype ALD-ODS.

## Results

Sixty-four caregivers of children living with HIV between 5 months and 13 years of age participated in four of the FGDs and 25 healthcare providers (HIV, pediatric, pharmacists, nurses, mentor mothers) participated in three FGDs. Most caregivers were female, parents, and living with HIV themselves. Similarly, most providers were female and averaged nearly 10 years of clinical experience. The average age of children interviewed was 9.3 years (5.4–14.9 years), 14 (58.3%) were male, 17 (70.8%) were aware of their HIV status, and all acquired HIV perinatally (average age at diagnosis was 1.8 years). Sociodemographic characteristics of caregivers, providers, and children are outlined in Table 2.

**Table 2:** FGD Participant Characteristics.

Caretaker Characteristics	N = 64
Age, mean [SD] years	38.2 [SD]
Sex	
Female	56 (87.5%)
Male	8 (12.5%)
Disclosure of child’s HIV status	
Disclosed to anyone	60 (93.75%)
Disclosed to no one	4 (6.25%)
HIV status	
Positive	47 (73.4%)
Negative	17 (26.6%)
Relationship to Child	
Parent	44 (68.75%)
Grandparent	13 (29.7%)
Aunt/Uncle	6 (9.4%)
Sibling	1 (7.8%)
Education Level	
Some primary	27 (42.2%)
Completed primary	19 (29.7%)
Some secondary	6 (9.4%)
Completed secondary	5 (7.8%)
Some university and beyond	7 (10.9%)
Average weekly income	
<500 Kes	21 (32.8%)
500-750	22 (34.4%)
750-1000	16 (25%)
1000-2500	4 (6.25%)
2500+	1 (1.6%)
Residence	
Rural	48 (75%)
Sub-urban	10 (15.6%)
Urban	6 (9.4%)
Provider Characteristics	N = 25
Age, mean (SD) years	35.4 [7.1]
Years of experience, mean (SD)	9.5 [4.8]
Sex	
Female	19 (76%)
Male	6 (24%)
Type of Provider	
Clinician (CO or Nurse)	10 (40%)
Mentor Mother/ Counselor	10(40%)
Pharm Tech	4 (16%)
Community Health Volunteer	1 (4%)
Provider Department	
Clinical Care Center (CCC/HIV)	15 (60%)
Maternal Child Health (MCH)	3 (12%)
Prevention of Mother to Child Transmission (PMTCT)	6 (24%)
Out-patient Department (OPD)	1 (4%)
Children Characteristics	N = 25
Age, mean (SD) in years	9.3 [2.2]
Sex	
Female	11 (42%)
Male	14 (58%)
Age at diagnosis, mean yrs (SD)	1.8 [3.2]

### Preferences and acceptability of ODS characteristics

ODS Flavors & Taste. Both caretakers and providers thought the pleasant taste of the ALD-ODS prototypes compared to syrups or crushed pills would offer a significant improvement to the experience of caretakers administering medication and children consuming the medication.

*“Sometimes that drugs is bitter so it will make the child fear the medication. But this one with the taste, the flavor, and the sweetness, I think adherence is going to be good.” (Provider 04)*

Among adult participants that rated all four flavors (n = 34 participants), sweet and mango were most highly ranked with mean [SD] of 3.06 [0.85] and 3.03 [0.83], respectively, on a scale of 1–4. While some people selected orange or strawberry as their favorite, mean scores of these flavors were much lower (2.41 [1.2] and 1.53 [0.89], respectively). According to the mean rankings, sweet flavor ranked the highest among all participants (3.04 [0.94] adjusted for all flavor rankings) and no one ranked it as least favorite. Using pairwise ranking among all participants, 48 preferred sweet over mango and only 40 preferred mango flavor over sweet. Among children, 21/25 chose to sample the sweet flavored ODS and 20/21 (95%) of children reported liking the sweet flavor and would prefer it to their current pill-based regimen.

Concerns regarding the taste of the placebo ALD-ODS included a bitter after taste of some flavors; artificial tasting flavors, or that too sweet of a flavor could result in stealing of medication. A few participants questioned whether the taste would change once the medication was added. Some people who preferred the mango flavor noted the somewhat bitter aftertaste may be helpful to retain the appreciation that the ALD-ODS are medicine rather than candy.

*But if it is for the children, it is better for them to have the mango flavor [described as having a somewhat bitter after taste] because the child will come to like the sugar flavor so much and even want a second piece. Because we have those children who can even steal the medicine. (Caregiver 04)*

Size of Strip, Strips per Dose, & Doses per Day. Strip size, number of strips per dose, and number of doses per day were seen as interdependent. Strip sizes were deemed acceptable to participants and there was consensus that the size of the ODS should align with the age of the child.

This *smaller one [very small] is good for 1–3 years, the next [small] is good for 4–5 and the largest in size [medium] is good for 7–8 years’ old. (Caregiver 02)*

Caregivers described how they would prefer a larger strip if that would permit a single strip per dose – especially for younger children (<2 years) for whom medication administration is more difficult. This was consistent with caregivers ranking the medium size ODS as most acceptable (mean 4.3 [1.09]) while providers ranked the small size as most acceptable (mean 4.3 [0.99]).

*It would have been better if we just have one strip even if it means making it longer so that they can take it once. (Caregiver 06)*

All the children interviewed were currently taking regimens that required between 1 and 8 tablets per day with most children taking 3 tablets once per day – usually at night. Caregivers strongly preferred a single dose per day to reduce the stress of administration for both the caregiver and the child, fit better with daily schedules, and allow night-only dosing to minimize the impact of any negative side-effects like nausea or fatigue. Caregivers indicated they would prefer to give multiple strips per dose, if it would allow for once daily dosing.

*I feel that whether the child is given two or three strips, they should be given once a day. I am insisting on being once a day because it is stressful to give them medication twice. (Caregiver 05)*

This resonates with the quantitative data that multiple strips per dose are still acceptable among caregivers and providers, mean *4.5 [0.93],* particularly if facilitates once daily dosing. Caregivers of older children who had switched to a once-a-day regimen felt that switching back to multiple doses of ALD-ODS per day may not be worth it.

ALD-ODS Dissolution and Adhesion. All strip sizes dissolved in under 40 seconds and adhered in the mouth until dissolved. Participants were satisfied with strip adhesion and dissolution to mitigate any choking risks for infants or small children but the largest strip (i.e., ‘medium’) that dissolved fastest was deemed most acceptable across caregivers and providers, mean 4.6 [1.03]. Providers noted that placing the ALD-ODS on a young child’s tongue (as opposed to the palate or inner check) could be problematic if they stick out their tongue and try to remove it. They recommended reminding caregivers to monitor children until the strip dissolves, which could be hastened by initiating breastfeeding or administering a liquid immediately afterward.

*Those below 1 year... love sticking their tongues out. So, I was thinking if the strip can just be stuck on their palates instead, and have it dissolved by itself. They won’t be able to remove it, once stuck on the palate. Let’s encourage the mothers to initiate breast feeding immediately after administering the strip. (Provider 01)*

While most children interviewed no longer required their medication to be broken or crushed before administration, multiple children preferred how it [placebo ALD-ODS] just dissolved, making it easy to swallow, and how they liked that “even without water, you can swallow it at anytime.” (Child 03)

This resonated with a comments from a provider who noted how a patient used to take the medication well until she was old enough for boarding school.

*She stopped taking it [ART] because she feared that other students will know of her status. But this one, she can take discreetly, it doesn’t need water to take the drugs with. (Provider03)*

It was also noted that ALD-ODS carried in a pack or purse, particularly when traveling away from home, would “not make funny noises when shaken” (Provider 01), which could preserve privacy for caregivers and children.

**Fig 1:**
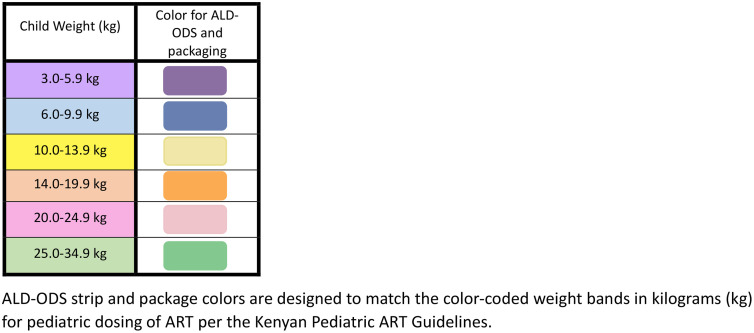
ALD-ODS Weight Band Color Coding. Caption: ALD-ODS strip and package colors are designed to match the color-coded weight bands in kilograms (kg) for pediatric dosing of ART per the Kenyan Pediatric ART Guidelines.

ALD-ODS colors and color coding. Participants found the colors (purple, blue, yellow, orange, pink, green) to be acceptable, see (Fig 1). Caregivers and providers both felt that the colors would be acceptable and attractive to children; means of 4.9 [0.47] and 4.8 [0.51], respectively. However, in general, medication color was not viewed as a very important characteristic. Caregivers noted color coding of ALD-ODS by weigh-based dosing would be helpful to quickly see the color of the strip and know that you are giving the right child the right weight-based dosage. This was deemed particularly helpful if a caregiver was administering medication to more than one child in the household or if they needed to seek the help of an alternate caregiver to administer the medication.

*The good thing about the colors is that assuming I am late to return home, I can call someone who is at home and ask them to give the child a particular color of medicine. (Caregiver 07)*

All providers felt that the use of color coding provided great clarity on dosing. They also noted that the colors were distinct and would be attractive to children. Providers highlighted that the use of color coding, i.e., matching the color of the strip and packaging to the color-coded weight bands for dosing as illustrated in Kenya’s National Pediatric ART Clinical Guidelines, (Fig 1) as beneficial for pharmacovigilance because it allows the pharmacist and caregiver to easily identify the appropriate box and dosage for the child.

Packaging: ease of opening. Caregivers were generally pleased with the ALD-ODS packaging options presented; one that required tearing the top of the individual ALD-ODS package or pealing back the two sides of the package to access the strip. Caregivers noted the benefits of the small, discreet size of the individual ALD-ODS packages that were easy enough for adults to open, but difficult for small children.

*The only problem is that if the child wants to use it and you are far away, it will be difficult for the child to open it. But if the parent is there and you have already learnt how to open it, then it will be less difficult. (Caregiver08)*

There were a few caregivers who struggled to open the packaging and suggested it be simplified by adding an “open here” indication or serration. Caregivers ranked ease of opening the package lower than providers, means of 4.1 [1.39] compared to *4.8* [0.52]. A few participants expressed concerns that if the user pulled apart the corners too vigorously, the medication may fall. For tearing, some worried that (1) users would accidentally tear the strip while trying to tear the packaging or (2) even after tear, you would have to reach into the package to retrieve the strip and this could be unhygienic.

The lack of a child safety lock mechanism for accessing ALD-ODS strips compared to other medications was raised as a concern, noting “*the other pediatric bottles come with a child lock. …But this one [ALD-ODS], the child can struggle and manage to open the strip. So, I think there is need to advise the guardian to store them safely.” (Provider02)*

Providers also noted the importance of counseling caregivers and older children to ensure that their hands were dry after washing before handling the strip; including a warning to not exceed the recommended dose nor dissolve the strip in water before taking it.

Bulk storage and dispensing. Providers found storage options satisfactory and preferable over the tins used for current formulations. Providers suggested that the strips come prepackaged in 30-, 60-, and 90- count boxes because boxes are easier to stack, shelve, and make dispensing easy. One provider highlighted that prepackaging ALD-ODS instead of storing them loosely in a box would simplify inventory and prevent misuse.

Quantitative rankings are displayed in Table 3 and integrated throughout the evaluation of ALD-ODS characteristics above. In summary, all characteristics were deemed acceptable. The ranking of ODS acceptability was similar between caregiver and providers on nearly all characteristics.

**Table 3:** Caregiver and provider quantitative rankings of ODS characteristics.

ODS Characteristic	Caregiver Score: Mean [SD]	Provider Score: Mean [SD]	Combined Adult Participant Score: Mean [SD]	Adjusted Mean Rank (flavor only)^3^ Mean [SD]
ODS flavors^1^				
Strawberry	1.7 [0.90]	*1.3 [0.89]*	*1.5[0.89]*	
Orange	2.8 [1.52]	*2.1 [1.18]*	*2.4 [1.2]*	
Sweet	2.8 [1.01]	*3.2 [0.83]*	*3.06 [0.85]*	*3.04 [0.94]*
Mango	2.7 [0.69]	*3.4 [0.85]*	*3.03 [0.83]*	*2.96 [0.94]*
ODS size^2^				
Very small	3.4 [1.68]	*3.5 [1.42]*	*3.4 [1.59]*	
Small	3.5 [1.25]	*4.3 [0.99]*	*3.8 [1.23]*	
Medium	4.3 [1.09]	*3.8 [1.30]*	*4.2 [1.18]*	
Strips per dose^2^	4.5 [0.98]	*4.4 [0.82]*	*4.5 [0.93]*	
ODS dissolution time (by size) ^2^				
Very small	3.4 [1.44]	3.5 [1.47]	2.6 [1.52]	
Small	3.3 [0.96]	4.3 [0.74]	3.6 [0.99]	
Medium	4.7 [0.83]	4.2 [1.34]	4.6 [1.03]	
Colors and color coding^2^	4.9 [0.47]	4.8 [0.51]	4.9 [0.48]	
Packaging: ease of opening^2^	4.1 [1.39]	*4.8* [0.52]	*4.3 [1.24]*	

^1^evaluated on a scale of 1 to 4, with 1 being least acceptable/preferred and 4 being highly acceptable/preferred.

^2^evaluated on a scale of 1 to 5, with 1 being least acceptable/preferred and 5 being highly acceptable/preferred.

^3^adjusted mean rankings of the top two flavors using a four point scale.

## Discussion

Participants felt the competitive advantages of ODS, most specifically ease of administration and pleasant flavor, could significantly reduce the chronic tensions experienced by both caregivers and children when administering current pediatric ART formulations [3,25]. Results from quantitative data support acceptability among caregivers and providers of at least one of the options offered for each prototype ALD-ODS characteristics (mean of 3.05 for taste on a scale of 1–4, and means between 4.2 to 4.9 on a scale of 1–5). While the children interviewed in this study had all transitioned to swallowing complete or split tablets, they preferred the option of ALD-ODS given the pleasant taste and ease and discretion of taking them when away from home. The need to strategically design individual ALD-ODS packaging that is easy enough for older caregivers with less nimble fingers yet resistance enough to keep young children from opening them was discussed. While viewed as a favorable innovation for people living with HIV of all ages, participants agreed that the unique characteristics of ALD-ODS held the greatest value for infants and children too young to swallow tablets.

Limitations. One of the limitations of this study was the inconsistency in evaluating ALD-ODS flavors due to a shipping delay of the orange and strawberry flavored strips. Children tried only sweet flavor of ODS, which was ranked highest among adult participants, thus offered general feedback on the taste rather than ranking data. Given the close ranking between sweet and mango flavored ALD-ODS, it may be worth additional taste testing of these flavors or considering offering a choice between these two flavors if deemed feasible. Even though ART drugs will be encapsulated for taste masking in the final product, flavors could potentially change slightly in the final, taste-masked, formulations. As some participants indicated, having a mild bitter or medicinal taste combined with the sweet flavor could have advantages to distinguish the ODS from candies.

Study staff engaged in FGD facilitation were residents in the regions where data collection occurred, spoke English, Swahili, and the local language. None of the US-based researchers (university or industry) engaged in FGD or interviews to avoid influencing participant responses. Despite robust discussion regarding the pros and cons of various ALD-ODS characteristics, there was a tendency to reach group consensus, which is consistent with more collectivists cultures [26]. This likely resulted in similar ranking among members of the same FGD, which appears to have been most evident for post-FGD ranking of ODS flavors. For practical reasons, the average age of the children interviewed was older than the age range of children most burdened by currently available ART options (0–5 years).

This study was strategically implemented in parallel with a small business innovation research (SBIR) grant to Oak Therapeutics (R43AI170237), allowing timely integration of user preference data to inform ALD-ALD-ODS design decisions to optimize acceptability, feasibility, and ultimately high ART adherence to improve pediatric HIV outcomes and quality of life.

## Implications for practice

Caregivers and providers expressed enthusiasm for the proposed innovation of ALD-ODS to improve ART adherence and ultimately, treatment outcomes for infants and children. Given the importance of early ART initiation and viral suppression in infancy to limit the size of viral reservoirs [27], and the added challenges of obtaining effective and affordable second line treatment options should treatment failure occur [28], the potential return on investment of effective infant and toddler friendly ART formulations is high. Potential time saving opportunities of ALD-ODS over standard formulations were also noted by health care providers and pharmacists. Newly available dispersible tablets for the preferred pediatric ART regimen, Abacavir and Lamivudine (120/60 mg) and Dolutegravir 10 mg are recommended in the Kenyan Pediatric ART guidelines. A 2024 publication documented the availability of dispersible tablets in over 80% of the 110 public health facilities surveyed in Kenya, also noting the stock out of at least one essential pediatric formulation in 40% of facilities [29]. Data regarding caregiver and child acceptability of dispersible tablets has not yet been published, but the challenges of readily available potable water and the need for the child to consume all the water containing the dispersed medications may still pose challenges, especially for infants and young children.

## Conclusion

Providers and caregivers described multiple advantages of the ALDS-ODS prototypes over traditional ART regimens for pediatric ART, noting these advantages most relevant for infants and young children too young to swallow tablets. Characteristics of the presented ALD-ODS prototypes were found acceptable and mitigated multiple existing barriers to adherence. Nuanced preferences and recommendations will inform product design to optimize product adoption and utility.

## Supporting information

S1 File Inclusivity-in-global-research-questionnaire. (DOCX)
